# Giving voice to children in research: The power of child‐centered constructivist grounded theory methodology

**DOI:** 10.1002/nur.22231

**Published:** 2022-04-28

**Authors:** Indu Sudarsan, Karen Hoare, Nicolette Sheridan, Jennifer Roberts

**Affiliations:** ^1^ School of Nursing Massey University Wellington New Zealand; ^2^ Elderslea Rest Home, Oceania Healthcare Wellington New Zealand; ^3^ School of Nursing Massey University Auckland New Zealand; ^4^ Child and Youth, Greenstone Family Clinic Auckland New Zealand; ^5^ College of Healthcare Sciences James Cook University Townsville Queensland Australia

**Keywords:** asthma, children's voices, child‐sensitive methods, grounded theory, social constructionism

## Abstract

There has been a growing interest in giving voice to children in response to the introduction of the United Nations Convention on the Rights of the Child and evolving sociological discourses on childhood. Using child‐sensitive methodologies such as constructivist grounded theory (CGT) enables children's voices to contribute authentic, meaningful, and eventually more actionable data, capable of informing policies and practices in children's best interests. In this article, we discuss how researchers using CGT can privilege children's voices through effective knowledge coconstruction by creating a child‐sensitive research space and using methods that are appropriate to their abilities and interests. We draw on selected data from the first author's (I. S.) PhD project that explores Indian immigrant children's and their family carers' beliefs, practices, and experiences of asthma in New Zealand. We encourage researchers to consider CGT as one of the appropriate methodological choices to explicitly promote the voice of the child.

## 1 INTRODUCTION

There has been a burgeoning interest in giving voice to children in recent years in response to the introduction of the United Nations Convention on the Rights of the Child (UNCRC) and evolving sociological discourses on childhood (Cudjoe et al., 2021; Cuevas‐Parra et al., 2020; Eastham & Kaley, 2020; Lundy, 2018). These models portray children as active and competent social actors who have the right to be engaged in decision‐making concerning their lives. Children are thus considered capable of influencing societal issues and policies affecting them. These contemporary models of childhood emphasize the significance of researching *by* and *with* children rather than *on* them, by listening to their words, emotions, actions, and social interactions. Moreover, it has been argued that without their voice, understanding the lives of children would be incomplete (Eastham & Kaley, 2020; Stirrup, 2017).

A social constructionist perspective builds on the principle that children's beliefs and their knowledge about the world are actively constructed through their interactions with one another and their surroundings (Berger & Luckmann, 1991; Freeman & Mathison, 2009). These constructions are context‐specific and cannot be comprehended without a thorough analysis of society, history, and culture. Social constructionists, therefore, challenge the widely held belief that children experience life in a universal manner and claim that their experiences become meaningful when considered in light of multiple contexts (Charmaz & Thornberg, 2021; Greig et al., 2013).

Understanding childhood as a social construction also has an impact on how researchers approach children and position them in the research process (Mah et al., 2020; A. Smith, 2011). Researchers who follow social constructionist principles view children as competent individuals who can provide researchers with ideas and knowledge, and who deserve to be informed and respected. Moreover, the inclusion of children's voices in research is considered essential to elicit rich, first‐hand data on their experiences and perspectives. Supporting children's voices is a challenging and multifaceted task, however. Researchers should undergo a significant paradigm shift in their attitudes, behaviors and thinking if they are to genuinely support children's voices (Eastham & Kaley, 2020; Peters & Kelly, 2015; A. Smith, 2011; Tay‐Lim & Lim, 2013). In tandem with these developments, there is a growing interest in choosing research methodologies tailored to the rights of children. Constructivist grounded theory (CGT) methodology is one such methodology that is appealing to researchers. CGT, developed by Kathy Charmaz, is an interpretive version of Barney Glaser's and Anselm Strauss's classic grounded theory (Charmaz, 2016; Farragher & Coogan, 2020; K. Hoare et al., 2017).

While there is a wealth of literature illustrating the wide range of approaches and methods that can be used in research with children, very little is published regarding the use of CGT as a child‐sensitive methodology. In this article, we begin by explaining relevant theoretical perspectives of childhood that have informed our argument in favor of using CGT when researching with children, followed by a brief overview of the first author's (I. S.) current doctoral study that required children's active participation. In the next section we discuss various rationales for selecting CGT as the methodology to privilege the voices of children for the current study. In the final section, we use selected data from I. S.'s doctoral project to critically analyze how in‐depth semistructured interviews employing drawing and photography facilitated engagement with children. This section also highlights the importance of ensuring child‐sensitive research spaces and techniques in promoting children's voices. Although family carers were also interviewed to provide insights into their child's experiences of asthma, the focus of this article is on how to include children's voices in research.

## 2 THEORETICAL PERSPECTIVES OF CHILDHOOD

Researchers' assumptions about children influence how they conduct research and the knowledge they produce. Children's voices can only be promoted by using methodologies that are based on theoretical frameworks that recognize and respect their assets, competencies, and developmental context (Mah et al., 2020; M. Smith et al., 2021). We draw on three important perspectives to describe the rights and theories of childhood. These perspectives are: the UNCRC, Vygotsky's sociocultural theory of cognitive development, and childhood studies (Peters & Kelly, 2015; R. Smith, 2013; United Nations International Children's Emergency Fund, 1989).

The UNCRC has significantly influenced children's participation in research as a means of exercising their participatory rights (Cuevas‐Parra, 2020). Introduced in 1989, the UNCRC consists of a set of universal standards that were developed to give voice to children and young people. Child participation is one of the core principles of the UNCRC, which advocates the rights of children and young people to freely express their views and participate in decisions on all matters affecting them (United Nations International Children's Emergency Fund, 1989). Articles 12 and 13 of the UNCRC demonstrate these rights:

Article 12 (respect for the views of the child) states:

Parties shall assure to the child who is capable of forming his or her own views the right to express those views freely in all matters affecting the child: the views of the child being given due weight in accordance with the age and maturity of the child. (United Nations International Children's Emergency Fund, 1989, p. 4).

Article 13 (freedom of expression) reinforces Article 12 and states:

The child shall have the right to freedom of expression; this right shall include freedom to seek, receive and impart information and ideas of all kinds, regardless of frontiers, either orally, in writing or in print, in the form of art, or through any other media of the child's choice. (United Nations International Children's Emergency Fund, 1989, p. 4).

Article 12 should be interpreted in conjunction with Article 5 of the UNCRC (Lundy & McEvoy, 2012). Article 5 points out the rights of adults in providing proper guidance and direction to the child which is consistent with the evolving capacities of the child. Adults' rights over the child are expected to gradually lessen and eventually stop as the child matures. The UNCRC acknowledges children's legal status as minors while also enabling adults to act as advocates, mediators, proxies, and interpreters as needed (Ford et al., 2018; Lundy & McEvoy, 2012; Mutambo et al., 2019). Therefore, the people with whom children develop close relationships play a key role in their life as they structure children's lives and give meaning and direction to their experiences. These relationships and the key role they have are commensurate with Vygotsky's sociocultural theory of cognitive development (R. Smith, 2013).

Vygotsky, a Russian psychologist, claimed that children actively construct knowledge through collaborative activity. He associated the influence of sociocultural environment to children's cognitive development and questioned the notion of universal child development by Jean Piaget (Greig et al., 2013). Piaget's staged account of a child's cognitive development emphasized how children constructed their perspectives of the world through individual activities. His theory received criticisms for restricting understandings of children's capabilities and ignoring the sociocultural influences that shape their development. In contrast, according to Vygotsky's sociocultural theory, a complex interaction of several social, cultural, and historical factors determines how children respond to various situations and may vary with person, place, and time. The focus of Vygotsky's theory is the role that social interaction plays in children's development (A. Smith, 2011). Social interaction contributes significantly to children's knowledge acquisition; they learn as they interact with their family members, friends, and other members of society. Importantly, children do not just copy what they see around them but actively attempt to make sense of it. At the same time, adults assist children in the development of their higher cognitive functions such as thinking and problem‐solving. Children's development is therefore coconstructed. The term coconstruction acknowledges children's active role as competent participants in their own development and adults as those with adequate expertise who guide them. Vygotskian views urge researchers to consider the quality and nature of the child's environment, age, social relationships, culture, and experiences while interpreting their perspectives (Eastham & Kaley, 2020; Greig et al., 2013).

Childhood studies emerged as a critique of the portrayal of children as a social minority group who lack independence, confidence, intelligence, rationality, and autonomy (R. Smith, 2013). Childhood studies have substantially contributed to recognizing children's agency and social construction of childhood. Agency refers to the ability of children to comprehend, actively participate in, and influence the world around them, demonstrating competence in all matters affecting their wellbeing (Cudjoe et al., 2021; Ford et al., 2018; Mutambo et al., 2019). Furthermore, childhood is viewed as a social construct that changes through time based on values, beliefs, philosophies, and cultures, even within the same society. The way society constructs childhood can have a significant impact on how children participate in various aspects of life. Importantly, with the evolution of childhood studies, the concept of childhood is embraced in a way that ensures the best interests of children and affirms that children's voices are not ignored or that the access of dominant discursive realms to them is not limited. For example, children who were previously classified as highly vulnerable, such as those with chronic medical conditions, are now considered knowledgeable about their illness experience. It is argued that these children's viewpoints can only be elicited from them, and their viewpoints are likely to differ from those of their adult caregivers who usually talk on their behalf (Mah et al., 2020; Mutambo et al., 2019).

## 3 THE DOCTORAL STUDY

Childhood asthma is one of the major health issues among Indian immigrant children in New Zealand (Mehta, 2012; Scragg, 2016). Despite the lower prevalence rates compared to other minority ethnic groups, the potentially avoidable hospitalization rates due to asthma among Indian immigrant children are higher than their New Zealand counterparts. Moreover, asthma is the first leading cause of potentially avoidable hospitalization among this group whereas dental conditions and ear, nose, and throat infections are placed second and third, respectively (Mehta, 2012; Plunket, 2015; Wong & Tsang, 2018). Despite the steadily growing population of Indian immigrants in New Zealand, little research has been conducted on childhood asthma. In contrast, among the limited international studies on childhood asthma, most of the studies focus on the experiences of family caregivers and health professionals; relatively few studies examine children's perspectives.

New Zealand ratified the UNCRC in 1993 and is obliged to align the laws and policies of the country to the convention's standards which are in the best interests of children (Dalli & Stephenson, 2010). The progress of incorporating the UNCRC into policies, laws, and processes, however, has been slow over the last 25 years (The Children's Convention Monitoring Group, 2018). Additionally, one of the priority child rights issues identified by the UNCRC Monitoring Group in New Zealand is “Piecemeal approach to gathering the views of the child” (UNCRC Monitoring Group, 2015; p. 1). The Ministry of Vulnerable Children (2017) is therefore actively seeking the input of experience of children and young people to improve the current health services.

The underrepresentation of Indian immigrant children with asthma in research has led to a discrepancy in knowledge to inform practice. As children are the only ones who can provide the most accurate information about their experiences, it is imperative to listen to their perspectives regarding their experiences of asthma. However, in some cases, it is equally important to consider the adults' perspectives on their child's condition to gain a comprehensive picture of the situation. For example, Indian family caregivers' beliefs on chronic conditions such as asthma shape the context in which they provide care to their children (Lakhanpaul et al., 2019; Mehrotra et al., 2014). Moreover, Indian children often identify themselves in reference to the people around them and the social circumstances they find themselves in. Change can only occur with the help of those with whom the child interacts the most. This may be because of their collectivistic cultural orientation where familial, and community preferences are given more importance than individual preferences (Benuto et al., 2014).

The challenges of including children in decision‐making and making their voices heard are acutely marked in the Indian context (Chadda & Deb, 2013; Raina et al., 2020). Children are considered inferior to adults who have the absolute authority to make decisions on their behalf. Adults also do not expect children to challenge their decisions. Additionally, in authoritarian parenting, which is popular among Indian families, children are expected to obey, depend on, and work together with parents and other elder family members in all matters affecting them. Parents may use a system of strict guidelines to develop, regulate, and evaluate their children's behavior. While authoritarian parenting does not imply a lack of compassion on the part of the parents, it emphasizes the culturally rooted nature of Indian parenting, which can create a power imbalance between parents and children. Indian immigrant children's power to participate in research, contribute meaningfully, and influence decisions that affect their lives are highly influenced by their parents or family carers (Kuppens & Ceulemans, 2019; Sondhi, 2017). Therefore, eliciting Indian family carers' insight into their child's asthma experiences and their experiences of being a carer to a child with asthma are extremely important.

According to Mah et al. (2020), there is no problem in relying on adult perspectives, nor is it problematic to combine adult‐generated with child‐generated data. The reliance on adults' views becomes problematic when their observations are used instead of children's own voices as the only evidence of a child's experiences. Thus, I. S. framed the study's central aim as exploring the beliefs, practices, and experiences of asthma among Indian immigrant children and their family carers.

### 3.1 CGT: A child‐sensitive methodology

To answer the research question, the authors required a methodology that enabled exploration of the social construction of childhood and that acknowledged its complexity, plurality, multiplicities, and diversities. This demanded a methodology that was open and flexible so that the child participants could gain ownership and control over the research process (Kyritsi, 2019; Poku et al., 2019). CGT as an interactive methodology was chosen and data were obtained through in‐depth semistructured interviews. There were other specific rationales for choosing CGT. First, CGT was chosen as it is best suited to study a topic about which little is known. Second, CGT is an appropriate methodology to explore interactions between people and their social settings and to investigate the complex social processes involved. For example, CGT has been proven as an excellent methodology for studying the experiences of people who are negotiating a new social environment such as immigrants or who are in the process of adapting to a recently diagnosed chronic illness. Finally, CGT can be used to undertake social justice inquiry and to investigate the relevant established impressions afresh (Birks & Mills, 2015; Charmaz, 2014; Oktay, 2012).

In CGT, children take a more active part in the coconstruction process, collaborating with researchers to select those aspects of their experience that are most meaningful to them. Therefore, CGT gives authority and respect to the voices of children (Abma & Schrijver, 2019). The key to knowledge coconstruction is the concept that the lived experiences of both children and researchers always influence the knowledge generated throughout the research. Hence, CGT assumes that researchers cannot completely set aside or bracket their knowledge and experience and can be utilized in the coconstruction of knowledge during the research (Charmaz, 2016; K. J. Hoare et al., 2012b; Mah et al., 2020).

In the current study, IS occupied a complex position in relation to studying Indian immigrant children's asthma experiences. Being an Indian immigrant parent in New Zealand and a former Indian pediatric nurse, she occupied an insider (emic) position. Moreover, she knew many Indian immigrant children with asthma in New Zealand through social networking. However, she was also an outsider (etic) as she did not have any children with asthma or had nursed any Indian immigrant children with asthma in New Zealand. I. S. reflected on her position as the researcher, coconstructing knowledge with the participants and how she could potentially move along a continuum between an emic and etic researcher at different stages of the research process (Charmaz, 2016; K. J. Hoare et al., 2012a). Reflection not only entails exploring the researcher's assumptions, knowledge, and experiences about the phenomena under investigation but also determining how these factors influence their interpretation of those phenomena and interactions with participants. Listening to children's needs based on self‐reflection and coconstructing children's experiences through shared interaction helps to address the power imbalances between the adult researchers and children, facilitating children's genuine involvement in research (Poku et al., 2019; Roa, 2019).

To foster reflexivity, I. S. had also been recording her thoughts in the form of memos from the early stages of the study. Memoing is an integral part of grounded theory and is an informal written record of the researcher's thoughts on the collected and analyzed data. Memos explain *how and why decisions* for all the actions taken associated with research and thus they stimulate analytic thoughts and are the key to conceptualization (Birks & Mills, 2015).

## 4 RESEARCH DESCRIPTION

Ethical approval was granted by Massey University Human Ethics Committee. The study sample included Indian immigrant children (8−17 years old) and their primary caregivers. This age range was chosen based on the evidence that research with children aged 7 years and older is most effective in eliciting meaningful data (Eastham & Kaley, 2020). The recruitment occurred through multiple channels based on the inclusion and exclusion criteria (see Table 1); these included general practices and schools from selected suburbs of Wellington and through various Indian cultural associations.

**Table 1:** Inclusion and exclusion criteria.

Participant type	Inclusion criteria	Exclusion criteria
Child	Indian immigrant children (8−17 years of age). Diagnosed with asthma for at least 1 year. Residing in New Zealand for at least 1 year. Able to speak and comprehend English. Able to understand what the research involves and give assent or consent to it.	Too unwell to take part. Having other long‐term illnesses (except eczema and allergic rhinitis). Children with food allergy as well as asthma.
Family carers	Have child or children (8−17 years old) with asthma. Able to speak and read English.	Unable to speak and read English.

Potential participants were given adequate time to consider whether they wished to participate in the study, allowing them to make a fully informed and voluntary decision. While the children had the option to select the venue for the data collection, the interviews occurred in an environment of the family caregivers' choice where they felt their children would be most comfortable, a potential reflection of the collectivistic nature of Indian families. Simultaneously, I. S. ensured that children were comfortable in the setting chosen by their family caregivers. The study initially employed a purposive sampling technique. In‐depth open‐ended semistructured interviews were conducted with children and their family carers which were digitally recorded and transcribed verbatim.

I. S. employed participatory data collection methods such as drawing and photography to facilitate interviewing younger children under 14 years. She was flexible in using these child‐sensitive data collection methods. For instance, some children preferred both drawing and photography, while others preferred neither. With the latter case, I. S. tried different methods based on the child's interests, such as storytelling and puzzles. Older children may consider these methods inappropriate for their age or babyish (Stirrup, 2017). Nevertheless, there was also the option for them to choose these child‐sensitive methods or any other if the situation deemed it necessary. Concurrent data collection and analysis took place accompanied by memoing and theoretical sampling.

Using theoretical sampling (Charmaz, 2014), I. S. was able to gain a deeper understanding of the participant's contextual and cultural interactions. For instance, during the initial stages of analysis, we realized that most children and their family carers were concerned by the negative judgment of asthma by others in the Indian community. Based on our analysis and follow‐up memos, we understood that social stigma had a major impact on the participants' asthma care decisions, thus making it an early potential category titled *fear of shame, blame, and discrimination*. We added new questions and probes to the future interviews to further explore this category using theoretical sampling. Further exploration of this category took place by selecting participants from diverse backgrounds, such as family carers with and without asthma histories, Indian children raised in New Zealand and children who migrated from India, younger and older children, and family carers with and without professional healthcare backgrounds.

## 5 GIVING VOICE TO CHILDREN THROUGH COCONSTRUCTION OF DATA

In CGT, the success of giving voices to children depends largely on the researcher's ability to employ the data coconstruction technique properly (Farragher & Coogan, 2020; Poku et al., 2019; Tay‐Lim & Lim, 2013). CGT researchers may, however, face challenges in coconstructing data with children, including implementing child‐inclusive techniques, and providing a child‐sensitive research environment. Researchers who fail to unlock the potentials of children as constructors, thinkers, and communicators within the construction process can disempower children, and tokenize their status as autonomous agents (Eastham & Kaley, 2020; Tay‐Lim & Lim, 2013). Hence, methods that provide children with shared control over language and concepts are critical for ensuring that their voices are heard effectively. To accomplish effective data co‐construction, children should be provided with the appropriate assistance they need to develop and express their opinions and should be placed in a social environment that fosters effective communication (Freeman & Mathison, 2009; R. Smith, 2013).

I. S. ensured an informal and flexible interview atmosphere for children to have their say. Special attention was taken to use developmentally appropriate vocabulary that makes sense for children to elicit knowledge about their beliefs, practices, and experiences about asthma. Children were not forced to answer questions or take part in activities that they did not want to do, and their verbal assent was sought on an ongoing basis. Additionally, verbal cues such as splutters, laughter, and nonverbal cues (eye‐contact, silence, gestures, body language, and facial expression) were also collected to best gauge their response to different questions and to monitor their interest in the interview (Carter & Ford, 2013; Due et al., 2013; Peters & Kelly, 2015; Webber‐Ritchey et al., 2021).

The duration of the interview varied with each child depending upon their attention span. Attempts were made to engage children by diverting them to another activity or a different topic if they seemed to lose interest in the conversation. If these attempts failed, interviews were terminated pleasantly. The interview was also paused as required; sometimes the child got distracted with other children in the house or seemed to have nothing more to contribute. I. S. invited family carers to accompany the children throughout the interview because she felt their presence would help to balance the researcher−participant power dynamics. Furthermore, if the interview occurred at home, not giving children the opportunity to have their parents present seemed inappropriate. While the carers' presence may enhance the child's narrative as they can assist in memory recall of events, it may restrict some children from expressing their views freely as there is a high chance that they can potentially dominate during the interview and the children's voices may remain unheard (Melton et al., 2014; O'Reilly & Dogra, 2016).

To minimize interruptions during the interview, family carers were informed of the objective of interviewing children separately to allow them to give their opinions and to listen to their voices. Despite this effort, there were interruptions during the interview when parents disagreed with children contradicting or correcting them or vice versa, making it more challenging for the children's voices to be heard. Interestingly, interviews conducted in children's homes provided the opportunity for other family members to add their thoughts and comments after the interviews. These incidental interactions provided valuable information about their attitude towards the child's condition and their asthma care experiences with the child (Eastham & Kaley, 2020; Melton et al., 2014).

### 5.1 Drawing and photography

Reflecting on the drawings and photographs at the start of the interview enhanced coconstruction beyond what was possible with interview methods alone, allowing for a richer and more complex understanding of children's experience of asthma (Mah et al., 2020). Children chose what they wished to draw about their asthma and were given the option of guiding discussions about it. Most children enjoyed these techniques which facilitated the establishment of rapport between I. S. and the children. I. S. did not employ any strict guidelines when it came to discussing their drawings. While some children talked as they drew, others talked about their pictures once they finished. Drawing enabled the elicitation of either abstract or tacit information. Some examples from the study included seemingly mundane activities and emotional insights that were difficult to elicit. Additionally, these techniques allowed I. S. to focus on what was important to them during their illness journey (Driessnack, 2006; Mah et al., 2020; Poku et al., 2019).

The study also included photography, in which children were encouraged to photograph anything about their asthma experiences that they felt significant. This method acknowledged children's agency as it allowed children to take photographs of their own choice and to lead the conversation based on the photographs they produced. As children are the most knowledgeable about the photos since they are the ones that owned them, they are more likely to speak confidently about their pictures and lead the conversation, ensuring a more effective coconstruction process. (Poku et al., 2019). However, I. S. was conscious of the potential influence of family members and friends on the capturing of the photographs as they may guide them about what to take or which photographs to be shared for the research.

**Figure 1:**
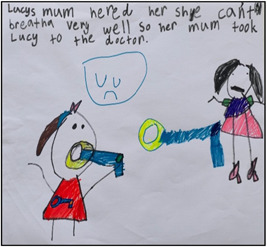
Drawing by Nidhi. [Color figure can be viewed at wileyonlinelibrary.com].

**Figure 2:**
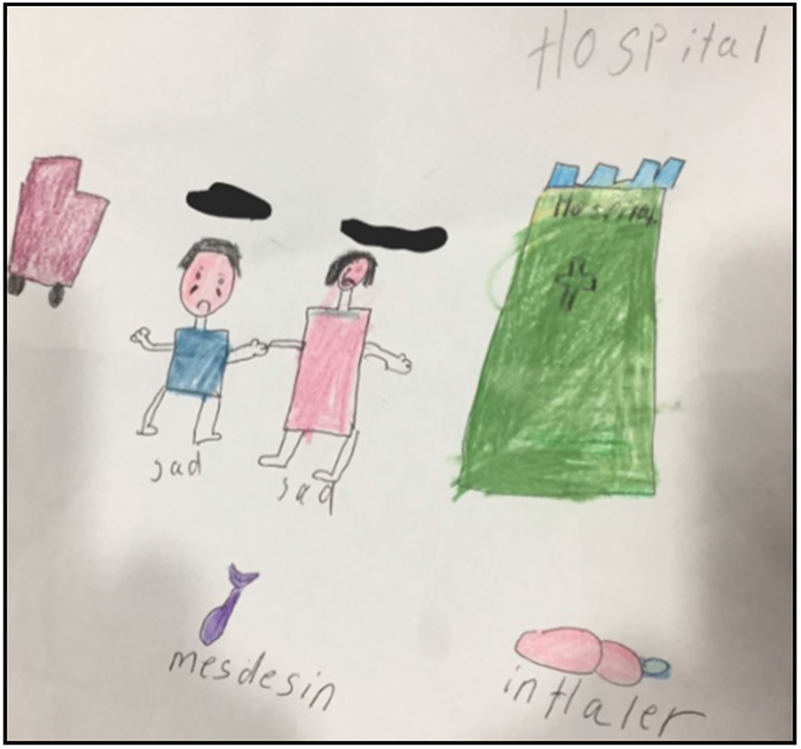
Drawing by Krishna. The name *Lucy is a made‐up name by the participant. [Color figure can be viewed at wileyonlinelibrary.com].

While data collection methods such as interviews demand timely responses, creative activities such as drawing and photography may be more leisurely, allowing children to think more critically about the response that they provide (Freeman & Mathison, 2009). As the proverb says, *A picture paints a thousand words*, some pictures yielded a comprehensive idea of children's asthma experience (see Figures 1 and 2). Pseudonyms are used for participants' names throughout the article to protect their anonymity.

**Figure 3:**
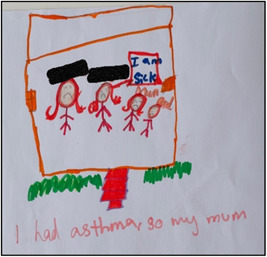
Drawing by Sruti. [Color figure can be viewed at wileyonlinelibrary.com].

**Figure 4:**
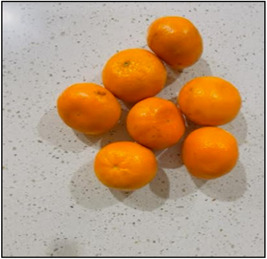
Photograph by Shikha. [Color figure can be viewed at wileyonlinelibrary.com].

During subsequent discussions about photographs/drawings, I. S. was able to capture their excitement, which often led to unique data regarding the children's asthma experience; their symptoms, how they felt emotionally, as well as their support network and management tactics. For example, Sruti's picture (see Figure 3) exemplified the vital role her family played in supporting her during asthma flare‐ups. Additionally, sometimes the most mundane images ignited valuable discussions that I. S. did not anticipate (Carter & Ford, 2013; Due et al., 2013). For instance, the image of oranges (see Figure 4) was taken by Shika, a 13‐year‐old girl, who had been eating oranges daily as a part of her mother's asthma control strategy. The photograph prompted an in‐depth and lively conversation about various natural remedies that Indian immigrants commonly used to manage asthma.

As mentioned earlier, without children's explanations, the images would have been meaningless (Peters & Kelly, 2015). By giving children the option to draw or photograph what they like and allowing them to have their say on it, they become an active part of the coconstruction process. Furthermore, when analyzing visual data, researchers run the risk of interpreting the data that are not congruent with what the children meant. The need to obtain an explanation from the child before the researcher interprets the visual data was repeatedly demonstrated during the project (Driessnack, 2006; Due et al., 2013). The following excerpt is taken from the interview with an 8‐year‐old girl, Nidhi, who describes her drawing (see Figure 1).

Researcher: Mom took Lucy* to the doctor and the doctor gave the blue inhaler? Nidhi: Yes because we can't use the orange inhaler.Researcher: Why? Nidhi: Because that means you are feeling good. Researcher: Ok.

As can be seen in the excerpt, the child shared her thoughts about the preventer inhaler (orange inhaler) when she explained her picture; she mistakenly believed that preventer inhalers should never be used during acute flare‐ups. This was a significant finding which the nurse researcher might have missed if the child had not been given the option of discussing her picture. Allowing them to explain about their pictures/photographs also helped to redistribute power in the researcher‐child relationship (Mah et al., 2020).

**Figure 5:**
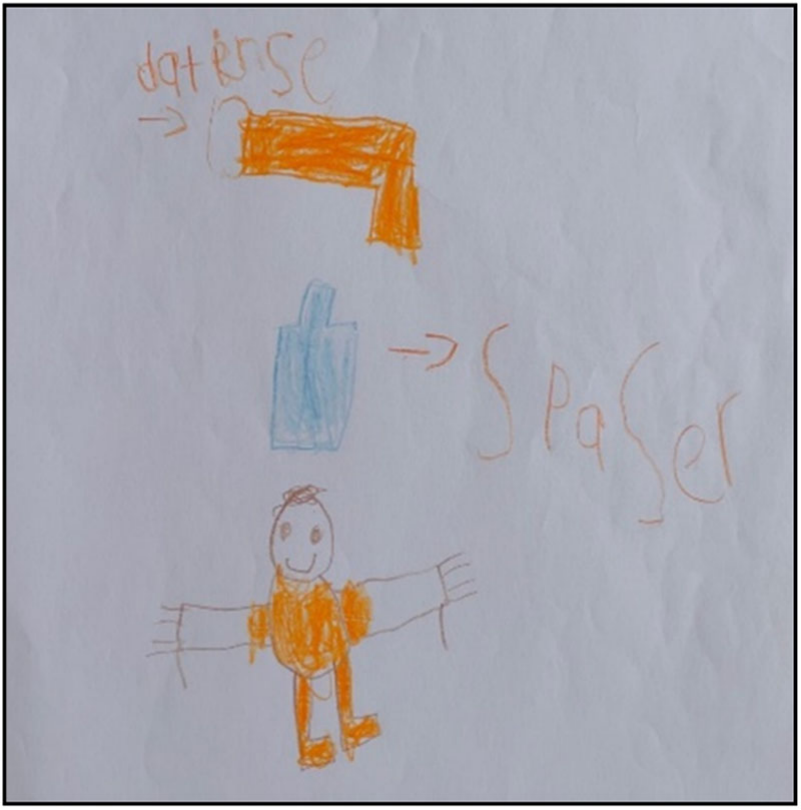
Drawing by Simon. [Color figure can be viewed at wileyonlinelibrary.com].

**Figure 6:**
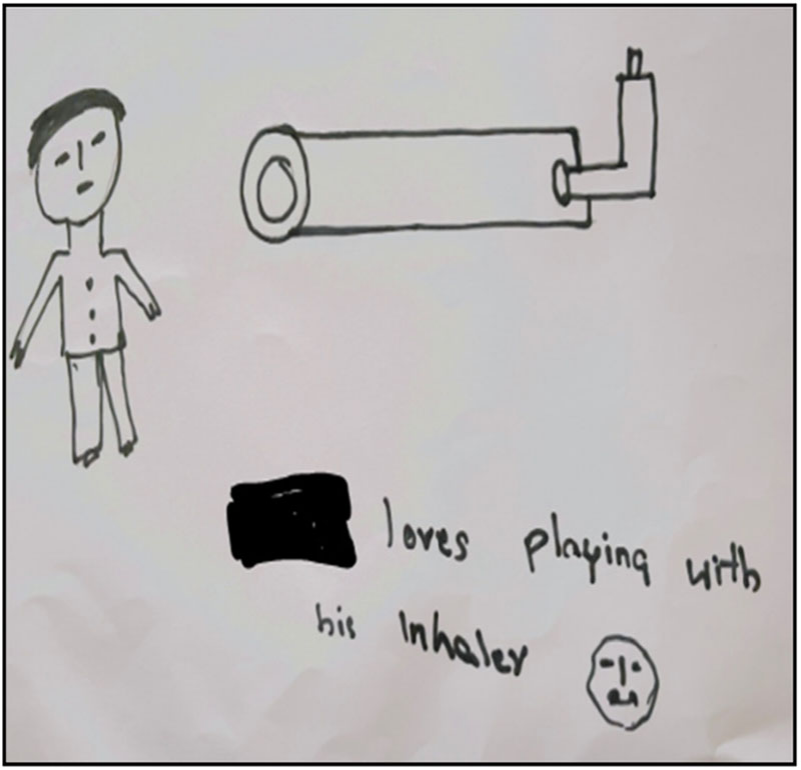
Drawing by Ryan. [Color figure can be viewed at wileyonlinelibrary.com].

I. S. also took time to clarify data collected from children as required since it was essential in ensuring rigor by enhancing the reliability of data collected (Due et al., 2013). Many children, for example, drew pictures of inhalers, and the stories behind each picture varied (see Figures 5, 6 and 5, 6). Simon drew a picture of a preventer inhaler (see Figure 5, 6). Through his drawing, Simon showed how the preventer inhaler, which he initially refused to use due to severe side effects such as painful mouth sores, had become his best friend. Simon claimed that he felt much better after starting the preventer inhaler, which was why he drew the happy face. Ryan too drew a picture of his inhaler (see Figure 5, 6), but the picture provided a different account; he explained how his mother had eventually consented to his choice of using the inhaler despite her preferences. Rekha, his mother, was originally hesitant to start the inhaler for her child because she was concerned about the adverse effects. Despite her constant attempts at various traditional asthma management methods, he refused to follow them and preferred to utilize the inhaler instead. During the interview, Ryan showed the researcher his inhaler decorated with stickers. He treated the inhaler as if it were a toy.

**Figure 7:**
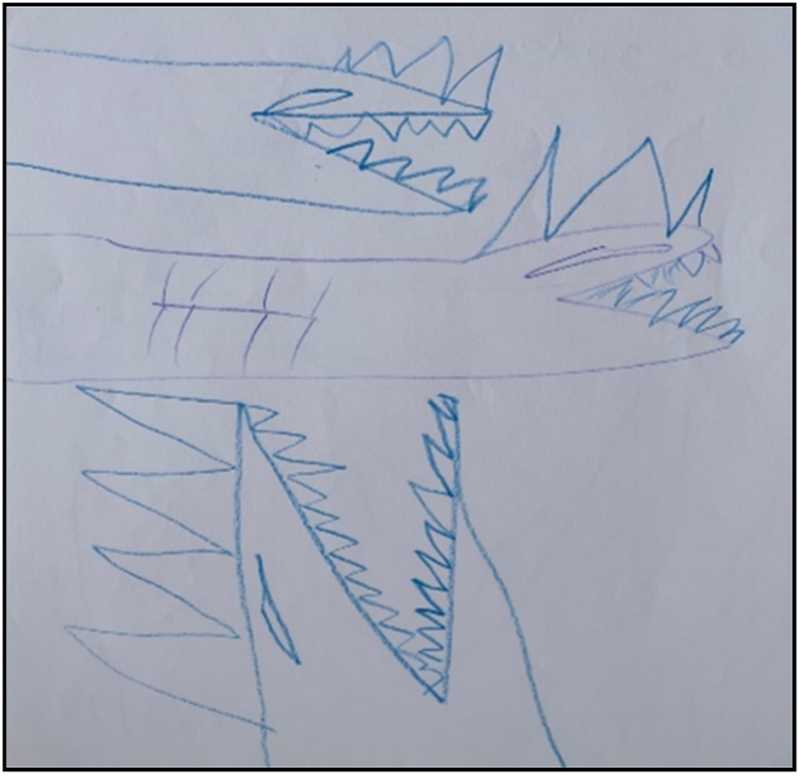
Drawing by Nehan. [Color figure can be viewed at wileyonlinelibrary.com].

There are, however, some drawbacks to using these techniques. As a researcher, one has no control over what images children might capture, so they may use the camera inappropriately, and some may consider taking pictures that have no relation with your research topic. In the case of drawing, a child may draw what is easy for them to portray. For example, Nehan drew a picture totally unrelated to the topic (see Figure 7). However, he was so interested in the activity of drawing that he wanted I. S. and his mother to guess what he was drawing when he was doing it. Nehan was shy at the beginning of the interview, but the drawing activity facilitated the establishment of rapport between us, and he took the lead throughout the whole interview process from the start.

On the other hand, the use of photographs as research data may present concerns about confidentiality, privacy, and copyright. To address this issue, children and their parents were informed of the purpose of using children's photographs, how it would be stored and used in the future, and how any identifiable features would be anonymized. Despite the drawbacks, these techniques are considered as an effective and quick way of obtaining a significant amount of information in a relatively short period of time (Fargas‐Malet et al., 2010; Poku et al., 2019).

## 6 DISCUSSION

Children are not a homogeneous group: they vary in their age, class, ethnicity, literacy, sexuality, cultural and religious background, level of articulacy and sociability, and the amount of adult assistance or monitoring they receive. These variables are interactive, dynamic, and complex (Freeman & Mathison, 2009). The same heterogeneity can be seen even within the same social or ethnic group which was evident in the current study sample of the Indian immigrant children. Charmaz's philosophy is consistent with the new sociology of childhood, which views children as contextually subjective, dynamic, and self‐determining beings. She claims that her position aligns with the ideologies of Lev Vygotsky, and emphasizes the role of social contexts, social interaction, interpretive understanding, and sharing viewpoints in exploring a phenomenon of interest (Charmaz, 2014; Greig et al., 2013).

Research with children has the potential to highlight surprising or unexpected findings which necessitate critical inquiry (Charmaz, 2016). While most qualitative research is inductive and emergent, CGT differs from other approaches and enhances critical inquiry in two ways. First, CGT integrates critical issues systematically into the analytic process. For example, in all versions of grounded theory including CGT, the researcher analyzes the initially collected data before moving to further data collection. Concurrent data collection and analysis also involves constant comparative analysis in which the researcher constantly engages with the data and compares each piece of data with others (Charmaz, 2014). Second, researchers who use CGT, design and adapt data collection to further explore avenues to pursue. For instance, the iterative data collection process which involves inductive, deductive, and abductive reasoning along with theoretical sampling ensures an in‐depth exploration of the phenomena of interest. While inductive reasoning involves arriving at generalizations based on the data collected, deductive reasoning involves exploring a known phenomenon or theory and determining if it makes sense in the given research context. On the other hand, abduction enables the researcher to promptly address and investigate any contradictory piece of data. Abduction entails considering all potential theoretical explanations for the surprising findings and then evaluating those explanations against new empirical evidence from the field (Charmaz, 2016).

## 7 CONCLUSION

CGT methodology, using child‐sensitive data collection methods, enables children's voices to contribute authentic, meaningful, and eventually more influential data capable of informing policies and practices in children's best interests. A CGT approach was chosen to study the asthma experiences of Indian immigrant children and their family carers and to develop an interpretative understanding of their beliefs, practices, and experiences within their complex social background. In this article, we have discussed how CGT researchers can privilege the voices of children through effective coconstruction of knowledge by creating a child‐sensitive research space and using methods that are appropriate to their abilities and interests. We have also highlighted the significance of gaining a comprehensive understanding of children's physical, social and cultural circumstances to promote their health and well‐being. These authors encourage researchers to consider CGT as one of the appropriate methodological choices to explicitly promote the voice of the child.

## AUTHOR CONTRIBUTIONS

IS conducted the research as part of her doctoral studies. Both KH and NS provided significant input into the design of the interview guides. Under the primary supervision of KH, IS performed the data collection and oversaw project activities. KH, NS, and JR assisted IS with the data analysis. IS and KH conceived the topic for the manuscript. IS prepared the first draft. KH contributed substantially to the organisation and revision of the content of the initial draft. NS and JR made significant contributions to reviewing subsequent drafts. All authors have contributed substantially to all phases of the manuscript development process as well as approved the final version.

## CONFLICTS OF INTEREST

The authors declare no conflicts of interest.

## Data Availability

The data that support the findings of this study are available on request from the corresponding author. The data are not publicly available due to privacy or ethical restrictions.
